# Atrial Fibrillation after Cardiac Surgery:  Where are we now?

**Published:** 2008-11-01

**Authors:** Dimpi Patel, Marc A Gillinov, Andrea Natale

**Affiliations:** 1 St David's Medical Center, Austin, Texas; 2 Cleveland Clinic Foundation Cleveland, Ohio

**Keywords:** Atrial fibrillation, postoperative atrial fibrillation, coronary artery bypass, antiarrhythmic agents

## Abstract

**Objective:**

To review: 1) Pathophysiology of postoperative atrial fibrillation (POAF); 2) Risk factors for POAF; 3) Prophylaxis of POAF; 4) Treatment of POAF; and 5) Future directions.

**Methods:**

We searched the Medline database for articles published between January, 1966 to September, 2008. We used the following keywords: Atrial fibrillation, Postoperative atrial fibrillation, Coronary Artery Bypass, and antiarrhythmic agents. Additionally, we searched references from all relevant articles.

**Conclusions:**

POAF occurs in 25-60% of patients depending on the type of cardiac surgery performed. POAF generally occurs on postoperative day 2 or 3. POAF is associated with an increased risk of morbidity and mortality, and longer hospital stay. Prophylactic treatments reduce the likelihood of POAF. In patients who experience POAF, rhythm strategies should be used in those who are symptomatic and hemodynamically unstable. All other patients should be managed with rate strategies.

## Introduction

An estimated 800,000 cardiac surgeries are annually performed in the United States [1]. The most common complication after cardiac surgery is atrial fibrillation (AF) and/or atrial flutter (AFL). Approximately 25-40% of patients have postoperative AF (POAF) after coronary artery bypass graft (CABG) and 50-60% after valvular surgery. The incidence of POAF is the highest in patients who have CABG and concomitant valve surgery, reaching 62%. The lowest incidence of POAF is seen in patients after heart transplant. The increasing incidence of POAF is most likely a result of the greater number of older patients having cardiac surgeries [2,3].

POAF episodes predominantly occur on postoperative days 2-3 and 70% of all episodes occur within the first 4 postoperative days. Recurrence of POAF most often occurs on postoperative day 3. Approximately 60% of all recurrences occur within two days of the initial episode of POAF. Nevertheless, POAF can occur at anytime after surgery. The main cause of hospital readmission after early hospital discharge following cardiac surgery is AF [2-5].

While POAF is often considered both transient and "benign", it has been associated with congestive heart failure (CHF), three-fold higher risk of postoperative stroke and renal insufficiency [2,3].  Moreover, POAF results in longer ICU and hospital stays by an average of 1 to 4 days  POAF is associated with increased cost per patient ranging from $5-20,000 dollars [2-4].  The financial burden of POAF exceeds $1 billion dollars annually in the United States [3,4]. For these reasons, several trials have been conducted in an attempt to decipher the various aspects of POAF.  This paper will review the relevant literature concerning: 1) Pathophysiology of POAF; 2) Risk factors for POAF; 3) Prophylaxis of POAF; 4) Treatment of POAF; and 5) Future directions in POAF.

## Pathophysiology of Postoperative Atrial Fibrillation

At present, it is hypothesized that AF is initiated by ectopic beats predominantly originating from the thoracic veins. Reentry, increased automaticity, and triggered activity have all been postulated as mechanisms that can cause arrhythmogenesis from the pulmonary veins. Some studies have indicated that an electrophysiological substrate within the pulmonary veins allows reentry due to heterogeneous refractory periods and decremental conduction. The pathophysiology of AF involved reentry of multiple wavelets which circulate around the atrium. Other studies have indicated that automaticity is the potential mechanism because of dependence on adrenergic stimuli and the presence of dissociated autonomic rhythms within the pulmonary veins that are electrically isolated from the left atrium. It has been noted that soon after disconnection of the pulmonary veins from the left atrium, the cycle length of electrical activity within the pulmonary veins becomes longer, and eventually the tachycardia resolves suggesting that the muscle sleeves surrounding the pulmonary veins depend upon electrical input from the left atrium to maintain pulmonary vein tachycardia. Dependence on an external input and the response to calcium channel blockers are in accordance to triggered activity. It is likely that the mechanism responsible for the initiation and perpetuation of AF is multifactorial [6].

While ectopic beats arising from the pulmonary veins have often been the culprit for initiation of paroxysmal AF in non-surgical patients, the exact mechanism of AF after cardiac surgery has yet to be determined. At present, there is not a definitive explanation as to why some individuals develop POAF and others do not. Nevertheless, most episodes are probably initiated by triggers such as premature atrial contractions in patients with a conducive atrial substrate.

Several studies have suggested that a heightened sympathetic response predisposes a patient to developing AF. However, it is interesting to note that the highest sympathetic levels are found 24 hours postoperatively and that most episodes of POAF develop on day 2 or 3 [2,6,7]. Furthermore, atrial refractoriness is dependent upon both sympathetic and parasympathetic contributions; therefore, the likelihood that a single culprit is responsible for POAF is low. Enhanced vagal tone has also been found in nonsurgical patients who have AF. Either heightened sympathetic or vagal tone may increase the likelihood of AF [6,7].

**Figure 1:**
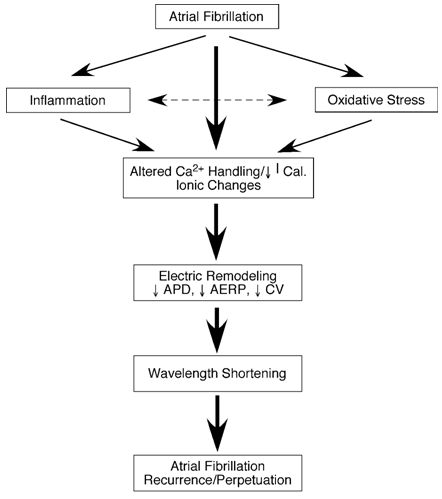
The potential role of inflammation and oxidative stress in atrial fibrillation-induced electrical remodeling.  I_cal_-L-type Ca^2+^ current; ADP-action potential duration; AERP-atrial refractory period; CV-conduction velocity.  Reproduced with permission from Korantzopoulos P, Kolettis T, Siogas K, Goudevenos J. Atrial fibrillation and electrical remodeling: the potential role of inflammation and oxidative stress. Reproduced with permission from Med Sci Monit. 2003;9:RA225-9.

The association between AF and inflammation was first observed by Frustaci who demonstrated that the atria of patients with lone AF had a higher prevalence of inflammatory infiltrates, myocyte necrosis, and fibrosis in comparison to patients without AF [8]. Since then, several studies have explored the role of inflammation in AF; however, there is still some debate as to whether inflammation causes AF. Rapid atrial activation causes an accumulation of calcium in atrial myocytes. As a result of the increased levels of intracellular calcium, there is a reduction of the inward L-type Ca^2+^ current. This results in both a shorter action potential duration and effective refractory period, thereby creating a favorable environment for the initiation and perpetuation of AF. In some cases the Ca2+ can cause cell apoptosis and subsequent inflammatory response and may contribute to structural remodeling and ultimately persistence of AF (Figure 1) [9].

C-reactive protein (CRP) is a highly sensitive systemic marker for inflammation. Elevated CRP levels have been shown to be a strong predictor of AF in both surgical and non-surgical patients. It has been shown that on postoperative day 2-3 the CRP levels are the highest, corresponding to the day of highest incidence of POAF. Moreover patients with a higher baseline CRP level are more likely to develop POAF after on and off pump surgery [10-12].

Oxidative damage has been found in the atrial myocardium of patients with chronic AF. The mechanism of oxidative damage in atrial myofibrils is mediated through the action of hydroxyl radicals and peroxynitrite leading to the formation of protein carbonyls and nitrotyrosines. Oxidative damage affects the myofibrillar creatine kinase which is an important controller of myocyte contractility which may contribute to severe contractile dysfunction and structural and electrophysiological remodeling [11]. A study that evaluated the effect of oral vitamin C on rapid atrial pacing induced atrial electrical remodeling in dogs provided further evidence supporting the role of oxidative stress in AF. In this study vitamin C was shown to eliminate ERP shortening. Additionally, vitamin C also inhibited the formation of nitrotyosine formation in atrial tissue, pointing to effective scavenging of peroxynitrite. Furthermore, this study also assessed whether prophylactic vitamin C reduced the incidence of POAF in patients who underwent CABG. This analysis suggested that in patients receiving prophylactic vitamin C the incidence of POAF was 34.9% in comparison to 16.3% in those that did not receive vitamin C. However, this was not confirmed by a prospective randomized study. It is speculated that vitamin C eliminates Ca^2+^ accumulation through scavenging reactive oxygen species that affect atrial proteins [12]. (Figure 1)

## Risk factors

Risk factors associated with POAF have traditionally been stratified as preoperative, intraoperative, and postoperative.  Several studies have identified characteristics that predispose a patient to POAF.  Nevertheless, the findings are often inconsistent between studies. Many prior studies have been conducted at a single center which limits the universality of their findings.  Inconsistencies can be attributed to the fact that patient characteristics varied between studies, utilization of a non-standardized method of identifying and defining arrhythmia, and small population sizes. In the future, if a uniform risk list can be accrued, it will provide insight into the pathophysiology of POAF, allow for prevention strategies, and help select patients who will benefit the most from such strategies [2-4].

### Preoperative

The incidence of AF increases with increasing age in the general population. Similarly, increased age consistently predicts a greater likelihood of POAF. With each decade of patient age, the incidence of PAOF increases by 75%. Atrial dilation, fibrosis and lipid deposits increase with age along with non-uniform anisotropic conduction. As a result of these histological changes, electrical conduction within the atria slows thus providing a substrate for arrhythmias. It has been hypothesized that increased amount of connective tissue and the resulting non-uniform anistrophic conduction peaks at 80 years of age and then plateaus. At present, few cardiac surgery patients are over 80 years old; however, the age of individuals undergoing cardiac surgery is increasing. The changes in the atrial substrate may not only be associated with the CABG surgery itself because a higher incidence of atrial fibrillation in older patients is seen in the non-surgical Framingham population [2,3,13].

Some studies have found an increased incidence of POAF in males, while other studies have reported no gender impact. The increased incidence of POAF in males versus females can be explained by differences in ion channel expression and by hormonal effects on autonomic tone.

Studies have shown that a prior history of AF increases the likelihood of developing postoperative AF. Mathew et al showed that 53% of patients with a prior history of AF had POAF [2].  Recurrence of AF can be due to alterations in the atrial substrate during surgery. However, it is more likely that the reason for recurrence is that preoperatively the patient has a diseased myocardium and with the surgery the patient is re-exposed to an environment that triggered the initial AF.

Recently, Osranek et al found that left atrial volume is a strong and independent predictor of POAF. Patients with a left atrial volume greater than 32 ml/m^2^  had an almost a 5 fold greater risk of POAF [14].

Several studies have found that prolonged preoperative signal averaged P-wave duration is a positive predictor for postoperative AF. Another study found that on surface ECG a prolonged PR interval or a prolonged P-wave duration identified patients with a higher risk for developing postoperative atrial fibrillation [15] [16].

Several studies have found that discontinuation of beta adrenergic antagonists prior to surgery increases risk of POAF Mathew et al have shown that preoperative withdrawal of either B-receptor antagonists or ACE inhibitors increases the likelihood of developing POAF to 50% and 46%, respectively [2].

Other patient characteristics associated with POAF include: COPD, CHF, rheumatic heart disease, right coronary artery stenosis, atrial ischemia, preoperative digoxin, left ventricular hypertrophy, elevation in left ventricular end-diastolic pressure prior to surgery, obstructive lung disease, and hypothyroidism [2-4].

### Intraoperative

Cardiopulmonary bypass deprives the heart of blood flow and thus could result in atrial injury and postoperative arrhythmias. Some studies have demonstrated that long procedure times can increase the likelihood of atrial fibrillation and that aortic-cross clamp time correlates with POAF [17-19]. Inadequate atrial myocardial protection during aortic cross clamping results in atrial ischemia triggering POAF. A study evaluated whether myocardial ischemic conditioning during surgery can prevent POAF. In this study 85 patients having on pump CABG were put into a control group or a group receiving 2 minutes of ischemic preconditioning achieved by temporary aortic cross clamping. The incidence of postoperative AF was lower in the ischemic preconditioning group (21.4%) than in the control group (46.5% P=.015) [18].

The location of venous cannulation has also been associated with POAF. Pulmonary vein and bicaval cannulation had also been associated with increased risk for POAF in some studies [19]. Furthermore, an elevation in intratrial pressure can increase the rate of waves coming from ectopic foci in the pulmonary veins in a stretch induced AF model [20].

Systemic hypothermia reduces metabolic demands of the ventricles and provides myocardial, cerebral, and somatic protection during the procedure. Traditional hypothermic cardioplegia does not adequately cool the atria or ensure complete electrical arrest in the atria. The use of warm cardioplegia is becoming common. However, studies of myocardial protection have not found different rates of postoperative atrial tachyarrhythmia associated with the various techniques [17,21].

Valve surgery is associated with an increased risk of POAF. The incidence of atrial fibrillation after valve surgery typically exceeds that in patients undergoing coronary revascularization alone, with the greater susceptibility believed to result from structural and hemodynamic abnormalities such as left atrial enlargement, pathological changes from rheumatic heart disease, increased left atrial pressure and surgical trauma [17].

### Postoperative

Pneumonia, chronic obstructive lung disease, hypomagnesaemia and prolonged ventilation are associated with atrial fibrillation after cardiac surgery. The need for postoperative atrial pacing is independently associated with atrial fibrillation. Moreover, it may also indicate underlying sinus-node dysfunction.

## Prophylaxis of Postoperative Atrial Fibrillation

### β-receptor antagonists (Vaughan-Williams class II)

**Figure 2:**
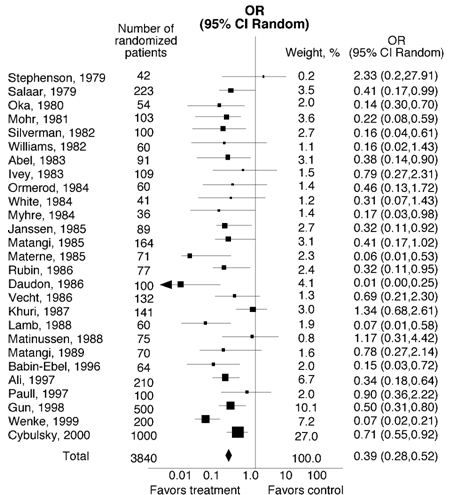
Beta blockers versus placebo or no treatment for the prevention of AF.  Test for heterogeneity P=0.00001.  Test for overall effect P<0.00001.  Published with permission from Crystal E, Connolly S, Sleik K et al.  Interventions on Prevention of Postoperative Atrial Fibrillation in Patients Undergoing Heart Surgery:  A Meta-Analysis.  Circulation 2002;106:75-80.

**Figure 3:**
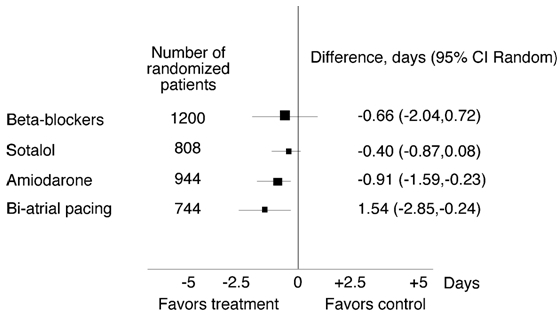
Effect of treatment on the length of hospital stay. Published with permission from Crystal E, Connolly S, Sleik K et al.  Interventions on Prevention of Postoperative Atrial Fibrillation in Patients Undergoing Heart Surgery:  A Meta-Analysis. Circulation 2002;106:75-80.

β-receptor antagonists (Vaughan-Williams class II) reduce the likelihood of developing POAF in many studies. Increased sympathetic tone may predispose a patient to POAF and β-receptor antagonists target this pathway. A meta-analysis of 27 studies evaluating 3,840 patients compared ß-receptor antagonist versus placebo for POAF prevention. (Figure 2).  There was a 14% reduction in incidence POAF between the control group and β-receptor antagonist group P<0.00001.   Furthermore, patients receiving long-term β -receptor antagonist therapy prior to cardiac surgery and who do not continue β-receptor antagonist postoperatively have a higher incidence of POAF. Therefore, unless specifically contradicted, reinitiation of β-receptor antagonists should not be postponed. Interestingly, two  β-receptor antagonist trials including 1200 patients showed no significant reduction in the length of hospital stay (-0.66 days; 95% CI, -2.04 to 0.72) (Figure 3). Trials have also compared β-receptor antagonists to digitalis, propafenone, and diltaziem. The findings from these studies were not conclusive due to  small sample size [2,22].

### Sotalol (Vaughan-Williams Class III agents)

Sotalol (Vaughan-Williams Class III agents) has a combination of β-receptor antagonist and potassium channel antagonist properties. A meta-analysis including eight randomized trials with 1294 patients compared the efficacy of sotalol versus placebo. The incidence of POAF in the placebo group was 37% and decreased to 17% in the sotalol group P<0.00001. However, 5 trials including 808 patients found that sotalol does not have any significant effect on the length of hospital stay (-.40 days, 95%CI, -0.87 to 0.08) (Figure 3).  Some limitations of the sotalol trials include: small subject size, exclusion of the sickest patients, and open label trials that might bias the results [2,16].

Since both sotalol and β-receptor antagonist are tantamount in terms of patient tolerability and side effects, a crucial issue is whether sotalol provides additional antiarrhythmic benefits for POAF offsetting the risk of associated proarrhythmia. Four trials compared sotatol with β-receptor antagonist and included a total of 900 patients. Two of the four trials showed statistically significant reduction in POAF in patients treated with sotalol and the larger of the two did not show significant reduction in POAF. When these four trials were directly compared via meta analysis, it was found that patients in the sotalol group had a 12% occurrence of POAF versus 22% in the β-receptor antagonist group (OR, 0.50; 95% CI, 0.34 to 0.74). Nevertheless while sotalol appears to be more effective than ß-receptor antagonist these trials are relatively small [2,22].

### Amiodarone

Amiodarone prophylaxis for POAF has been evaluated in 10 trials, including a total of 1,699 patients. Four trials found that amiodarone significantly reduced POAF. Nine trials including 1384 patients were compared via meta analysis. There was a 22.5% occurrence of POAF in the amiodarone group versus 37% in the control group P<0.00001. Five amiodarone trials including 944 patients (68% on amiodarone) reported a significant reduction in the length of hospital stay by 0.91 days (95% CI, -1.59 to -0.24) (Figure 3). Since amiodarone is associated with little risk of proarrhythmia, it can be used in patients with structurally abnormal hearts; however, only oral amiodarone has been shown to reduce POAF, and this requires a 7 day loading dose, which is not always feasible [2,22].

### Calcium Channel Blockers

There have been four trials conducted that evaluate the efficacy of calcium channel blockers including a total of 541 patients. Calcium channel blockers did not significantly reduce POAF in any of these trials [2,22].

### Magnesium

There have been 14 trials including 1,853 patients that evaluated whether magnesium chloride or magnesium sulfate supplementation reduced the occurrence of POAF. Only 1 of these 14 trials demonstrated a statistically significant reduction in POAF.  Although magnesium most likely does not offer prophylaxis against POAF, serum magnesium levels should be regulated [2,16].

### Digitalis

Ten studies including 1,401 patients have been performed assessing the efficacy of digitalis for the prophylaxis of POAF. The limitations of some of these trials include non-randomization and the fact that β-receptor antagonists were withdrawn postoperatively. Due to the limitations of these trials and the availability of better therapies, digitalis should not be used solely for prophylaxis of POAF [2,22].

### Atrial Pacing

Thirteen trials have been conducted to assess whether prophylactic pacing after cardiac surgery is able to prevent POAF. Overall, studies with prophylactic right atrial pacing and prophylactic left atrial pacing have been inconclusive. Bi-atrial pacing was found to reduce the length of hospital stay by 1.54 days (95%Ci -2.85 to 0.24) (Figure 3). Of the nine trials that assessed biatrial pacing versus control, POAF was reduced in seven trials. The inability to obtain consistent atrial capture throughout the postoperative phase appears to be an important limitation of this therapeutic modality. Limitations of these thirteen trials include different exclusion criteria, concomitant ß-receptor antagonist, and different techniques for recording AF episodes [2,22].

Meta analysis has shown that the incidence of POAF can be reduced by prophylactic therapy thus decreasing the length of hospital stay, risk of stroke, morbidity, mortality, and the cost of care. The ACC guidelines recommend ß-receptor antagonists be used as first-line prevention of POAF and that sotalol and amiodarone be used as second-line prevention [22,23].

AF prophylaxis reduces the length of hospital stay by 1.5 days. While this does not seem like a substantial reduction in length of hospitalization, only 50% of patients will experience POAF, thus, in order to detect a significant reduction in the length of hospital stay in an overall population the effect must be very large. Furthermore, with the use of prophylactic therapy there is a risk of side effects which influence the length of hospital stay. Regardless, even a small reduction in hospital stay can result in a considerable cost benefit especially since ß-receptor antagonists are inexpensive [2,22].

A meta-analysis including 2877 patients indicates that POAF prophylaxis has no statistically significant benefit in terms of stroke prevention [22]. The rationale is that most patients after cardiac surgery do not have POAF. Furthermore, if the likelihood of stroke with POAF is similar to that of chronic AF which is 5% per year, the risk of stroke during a 1 day episode of POAF would be less than 1 in 1000.

## Treatment of POAF

Much like non-surgical AF, the therapeutic issues in POAF center on restoring and maintaining sinus rhythm, controlling cardiac rate and preventing thromboembolic complications. Restoration of sinus rhythm should be pursued in patients with POAF who are hemodynamically unstable, symptomatic, or cannot tolerate anticoagulation. Rate management is appropriate for all other patients [24].

## Future directions in the treatment of POAF

New drugs such as a class III potassium channel blockers that block the rapid and slow I(kr) currents are promising. Trials are also under way assessing dronedarone, a drug that is similar to amiodarone [25].

The MAZE procedure is complicated and difficult to perform, therefore, it is not likely that it will become a mainstay for the management of POAF. Alternative approaches have become available and are increasingly performed. Atrial specific drugs, a better understanding of inflammation and prophylactic procedures in high risk patients may help limit health care costs, morbidity and mortality associated with the development of POAF.
